# Quality of life among post-menopausal women due to oxidative stress boosted by dysthymia and anxiety

**DOI:** 10.1186/s12905-016-0358-7

**Published:** 2017-01-03

**Authors:** Martha A. Sánchez-Rodríguez, Lizett Castrejón-Delgado, Mariano Zacarías-Flores, Alicia Arronte-Rosales, Víctor Manuel Mendoza-Núñez

**Affiliations:** 1 Unidad de Investigación en Gerontología, Facultad de Estudios Superiores Zaragoza, Universidad Nacional Autónoma de México, Av. Guelatao No. 66, Iztapalapa, CP 09230 México, D.F., México; 2 Hospital Gustavo Baz Prada, Instituto de Salud del Estado de México, Av. Adolfo López Mateos / Bordo Xochiaca S/N, Ciudad Nezahualcóyotl, CP 57300 Estado de México México

**Keywords:** Oxidative stress, Psychological disturbances, Quality of life, Menopause

## Abstract

**Background:**

Menopause is the onset of aging in women. During this process, some women experience physical changes that may impact upon their psychological and social status, also affecting their quality of life. Furthermore, several psychological changes following menopause have been shown to act as pro-oxidant, but the association between the psychological status that modify the quality of life and oxidative stress in postmenopausal women is still unclear. The aim of this study was to determinate the relationship between oxidative stress with psychological disturbances, low self-esteem, depressive mood and anxiety, and quality of life in the postmenopausal women.

**Methods:**

We carried out a cross-sectional study with101 premenopausal and 101 postmenopausal women from Mexico City. As markers of oxidative stress we measured plasma lipoperoxide levels, erythrocyte superoxide dismutase and glutathione peroxidase activities, and total antioxidant status. We calculate a stress score as global oxidative stress status, with cut-off values for each parameter; this score range from 0 to 6, representing the severity of markers modifications. All the women were rated using the Coopersmith Self-Esteem Inventory, the Zung Self-Rating Anxiety and the Zung Self-Rating Depression Scales, and the WHO Quality of Life-brief.

**Results:**

The postmenopausal women with low quality of life in the WHO Quality of Life-brief and their subscales had higher stress score compared with premenopausal women with high quality of life (*p* < 0.05). We found a positive correlation among lipoperoxide levels and Zung Self-Rating Anxiety and Zung Self-Rating Depression score (*r* = 0.226 and *r* = 0.173, respectively, *p* < 0.05), and a negative correlation with WHO Quality of Life-brief scores (*r* = −0.266, *p* < 0.01) in postmenopausal women. Multiple linear regression analysis revealed that average lipoperoxide levels increase by 0.0007 μmol/L for every 1-point increase in the Coopersmith Self-Esteem Inventory and by 0.001 μmol/L for every 1-point decrease in the WHO Quality of Life-brief, after adjusted for pro-oxidant factors. Zung Self-Rating Anxiety and Zung Self-Rating Depression Scales scores also contribute to increase lipoperoxides levels, but not significant.

**Conclusion:**

Our findings suggest that oxidative stress is increased in postmenopausal women with psychological disturbances and low quality of life.

## Background

In women, postmenopause is a critical period due to a series of endocrinological changes that are caused by the decline of production of estrogens by the ovaries (mainly estradiol) that lead to low estrogen levels. Although menopause is a natural process, some women experience physiological changes that may interfere with their ability to cope with their new psychological and social status and affect their quality of life [1, 2].

In this context, estrogen depletion affects many tissues of the body, including brain cells. Furthermore, the presence of steroid hormone receptors in various brain regions has been demonstrated, and it is known that estrogens increase cerebral blood flow, which prevents neuronal atrophy and enhances sleep, memory, cognition and other neurologic functions that are affected with low level of estrogens once menopause happens [3, 4]. The age-associated malfunction of human cells results from the physiological accumulation of irreparable damage to biomolecules, which is an unavoidable side effect of normal aerobic metabolism in a process termed as oxidative stress (OS).

Oxidative stress occurs when the antioxidant system is unable to effectively deal with the reactive oxygen species (ROS) and free radicals produced in living organisms. These ROS and free radicals then cause oxidative damage to biomolecules such as lipids, proteins and DNA, which leads to dysregulation of normal metabolism and physiology [5, 6]. Under normal conditions, ROS are cleared by antioxidants such as the enzymes superoxide dismutase (SOD), catalase (CAT), glutathione peroxidase (GPx), or by non-protein molecules [7], such as estrogens. These antioxidants are able to either prevent the generation of oxidizing species or reduced the oxidative effects of the ROS. As well as sex hormone, estrogens have free radical scavenging capacity present in the phenolic ring in A position and thus can function as antioxidant to inhibit the generation of ROS or neutralize excess ROS [8]. The evidence for the antioxidant activity of estrogens came from neurological, cardiovascular and metabolic fields [8, 9]; moreover, we previously suggested that depletion of estrogen in postmenopausal women could also cause OS [10]. Furthermore, most of the symptoms after menopause, such as insomnia, depressive mood and anxiety, are considered pro-oxidants [11–13] affecting the health and social status of menopausal women and therefore their quality of life. As we indicated previously, estrogens might have neuroprotective properties, such as controlling neuronal activity related to the process of cognition and the modulation of mood and other mental states, and improving learning and memory [9]; however, the association between the psychological status that modify the quality of life and oxidative stress in postmenopausal women is still unclear. Therefore, the aim of this study was to determinate the relationship between OS with psychological disturbances and quality of life in the postmenopausal period.

## Methods

### Study design and population

A community-based cross-sectional study was conducted in perimenopausal women from Mexico City, Mexico. They were invited to participate in the project “Menopause and oxidative stress” directed by the Gerontology Research Unit at Universidad Nacional Autonoma de Mexico, Zaragoza Campus, from November 2013 to May 2014. The baseline sample consists of 240 women recruited by advertisements placed near the university campus. The women were selected to establish two groups, premenopausal and postmenopausal. The main eligibility criteria for premenopausal women were that they had to be 40 to 52 years old; besides, they most still have menstrual period and they most experience menopausal symptoms, e.g. depressive mood, hot flashes, and sleep disturbances. For postmenopausal women, they had to be 48 to 59 years old and have at least 12 months of spontaneous amenorrhea and/or serum estradiol levels below 25 pg/mL and FSH levels higher than 50 mU/mL, both groups were free of cardiovascular and cancer diseases, without antioxidant supplement intake for at least six months prior to the beginning of the study, and without hormone therapy intake. Twenty-five women had no further interest in the study and seven were excluded because their age was more than 60 years old.

The selected women underwent the following examinations: complete clinical history, biochemical analysis, complete blood count, anthropometric and blood pressure measurements, after a 12-h fasting period. Those tests were used to establish their health status, and cut-off points for reference values for Mexican adults were determined at the Gerontologic Clinical Research Laboratory of the Universidad Nacional Autónoma de México, Zaragoza Campus in Mexico City [14]. The intra- and inter- assay variation coefficients of hematological and biochemical tests were less than 5% in all determinations. Also, we measured estrogens and FSH levels. The within-run precision levels for these methods were 3.1 and 7.4%, respectively, and the estrogens analytical sensitivity was 8 pg/mL.

### Sample size

The sample size was calculated based on a two proportion principle on the assumptions of a proportion of postmenopausal women (83%) and other of premenopausal women (56%), both with high lipoperoxides levels (LPO) and acute symptoms, as we previously reported [10]; using a 5% level of significance with a power of 90%. To detect a difference of 27% in the proportions, we estimated sample size approximated by the chi square test with Yates’s continuity correction. To compute the sample size, we used the tables for clinical studies [15]. We added 20% of the calculated sample size to compensate the expected non-response. The final size obtained was 190 women, but to ensure the results, we selected 101 randomized women by group.

### Oxidative stress measurement

We measured red blood cell superoxide dismutase (SOD) activity by the method of xanthine oxidase; red blood cell glutathione peroxidase (GPx) activity by the oxidation of glutathione; and plasma total antioxidant status (TAS) by 2,2-azino-bis (3-ethylbenzthiazoline-6-sulfonic acid, ABTS^+^) radical formation kinetics, all the assays with commercial kits (Randox Laboratories, Ltd., Crumlin Co. UK). Also we measured plasma lipoperoxides levels (LPO) with thiobarbituric acid reacting substances (TBARS) assay described by Jentzsch et al. [16]. All the methods were validated in our research laboratory and the within-run precision for the markers were as follows: 3.8, 4.6, 4.3, and 6%, respectively [10]. Artefactual formation of TBARS in the samples was prevented by adding 10 μL of 2-mM butylated hydroxytoluene (BHT) in ethanol at 95% immediately after centrifugation. The measures were performed in a Shimadzu UV-1601 UV–vis spectrophotometer (Kyoto, Japan).

In addition, we calculated SOD/GPx ratio, and antioxidant gap with the equation [17]:

$$ \mathrm{GAP} = \Big(\mathrm{T}\mathrm{A}\mathrm{S}\ \hbox{--}\ \left[\left(\mathrm{albumin}\ \left(\upmu \mathrm{mol}\right)\ \mathrm{X}\ 0.69\right) + \mathrm{uric}\ \mathrm{acid}\ \left(\upmu \mathrm{mol}\right)\right] $$

Alternative cut-off values of each parameter were defined on the basis of the 90^th^ percentile of young healthy subjects: LPO ≥0.320 μmol/L, SOD ≤1.20 U/gHb, GPx ≤50.1 U/gHb, TAS ≤900 μmol/L, SOD/GPx ≥0.023, GAP ≤190 μmol/L. A stress score (SS) ranging from 0 (no oxidative stress) to 6 (severe oxidative stress) was calculated as oxidative stress status; a score 1 was given to each value higher or lower than the cut-off.

### Assessment of psychological status and pro-oxidant factors

The women were rated using validated self-assessment questionnaires in the Spanish version, and a structured questionnaire about pro-oxidant factors. Two trained bachelor nurses were used as data collectors and a nurse with a master’s degree was assigned as supervisor, all the psychology tests were conducted and evaluated by a psychologist with a master’s degree, coauthor of this paper.

Self-esteem was evaluated with the Coopersmith Self-Esteem Inventory (SEI). The minimum score possible is 0 and the maximum score is 100 points. A cut-off value lower than 50 points was considered to indicate low self-esteem [18]. The Zung Self-Rating Anxiety Scale (SAS) was used to assess anxiety, the total score on the SAS ranges from 0 to 80. A cut-off value higher than 45 points was considered to indicate anxiety [19, 20]. Dysthymia was determined with the Zung Self-Rating Depression Scale (SDS), the score ranges from 20 to 80. A woman with a SDS score above 40 points was considered with dysthymia [21, 22]; and the World Health Organization Quality of Life, brief (WHOQoL-brief) served to evaluated the quality of life. This instrument includes four subscales (physical, psychological, social relationships and environment) and two global questions regarding quality of life and overall health [23]. A cut-off value less than 96 points in the total score, lower than 26 points in the physical subscale, lower than 23 points in the psychological subscale, lower than 12 points in the social relationships subscale and lower than 30 points in the environment subscale were considered to indicate low quality of life [24]. Finally, the participants answered a structured questionnaire about pro-oxidant factors as potential confounders. We considered a pro-oxidant factor present, for each, when the following were noted, smoking ≥ 2 cigarettes/day, consumption of ≥ 2 glasses/day alcoholic beverages, consumption of > 2 cups/day caffeinated beverages, < 30 min/day of physical activity, and sleep < 6 h/day.

### Data analysis

Data were expressed as the mean ± standard deviation of the health status variables, tests scores, and oxidative stress markers in pre- and post-menopausal women; these values were compared using Student’s t-test. Percentage differences were evaluated using the chi square test (χ^2^) and 95% confidence intervals.

Women were divided into four subgroups based on menopausal status and psychological disturbances to find the difference in stress score, as oxidative stress marker, 1) premenopausal women without psychological disturbances or with a high quality of life, 2) premenopausal women with psychological disturbances or a low quality of life, 3) postmenopausal women without psychological disturbances or with a high quality of life, and 4) postmenopausal women with psychological disturbances or a low quality of life. We considered a woman with psychological disturbances if she has dysthymia, anxiety or low self-esteem according to the respective cut-off value in the tests. The differences among the subgroups were analyzed by one-way ANOVA with the Dunnett test as post-hoc, using subgroup 1 as control group.

Pearson correlation analyses were calculated to study the association between the tests scores and oxidative stress, using LPO levels as oxidative stress marker, obtaining the relationship between each of the two groups, pre-and postmenopausal women. We selected LPO levels because they showed the biggest difference among all the oxidative stress markers according to descriptive analysis. Multiple linear regression analyses were performed to examine the association between LPO as dependent variable and the test scores as independent variables in both pre- and postmenopausal women. All the models were adjusted for number of cigarettes consumed and hours of sleep by day and BMI, as life-style pro-oxidant factors. Other life-style pro-oxidant factors were not included because they were very frequent (caffeinated beverages intake) or infrequent (alcohol intake and physical activity).

The level of statistical significance was set at a two-tailed *p*-value of 0.05. The data were processed by use of the standard statistical software package SPSS V. 20.0 (IBM SPSS Statistics Armonk, NY, USA).

## Results

### Sample characteristics

The biochemical-hematologic parameters, anthropometric and blood pressure measurements, and oxidative stress markers of the study participants are presented in Table 1. It was observed that women in both groups had similar values in all parameters, except in red blood count (*p* < 0.05). About of oxidative stress markers, LPO levels were higher and the activity of antioxidant enzymes were lower in postmenopausal compared with premenopausal women (*p* < 0.05).

**Table 1:** Baseline characteristics of women by study group

Parameter	Premenopausal women (*n* = 101)	Postmenopausal women (*n* = 101)	*p* value
Biochemical -hematologic parameters			
Hemoglobin (g/dL)	14.0 ± 1.5	14.6 ± 1.4	0.002
Hematocrit (%)	43 ± 3.9	45 ± 3.9	0.003
Erythrocytes (X10^12^/L)	4.6 ± 0.5	4.8 ± 0.7	0.034
Total leukocytes (X10^9^/L)	6431 ± 1573	6071 ± 1192	0.070
Glucose (mmol/L)	5.6 ± 2.5	5.2 ± 1.8	0.773
Urea (mmol/L)	9.6 ± 2.1	10.4 ± 2.5	0.085
Uric acid (μmol/L)	274 ± 77	280 ± 77	0.735
Creatinine (μmol/L)	72 ± 15	72 ± 12	0.848
Cholesterol (mmol/L)	5.6 ± 0.9	5.9 ± 1.1	0.057
Triglycerides (mmol/L)	2.0 ± 1.0	2.1 ± 1.1	0.552
HDL-c (mmol/L)	1.5 ± 0.4	1.6 ± 0.4	0.832
Estrogen (pg/mL)	101.7 ± 71.5	12.3 ± 6.2	<0.0001
FSH (mIU/mL)	10.7 ± 12.2	55.2 ± 26.1	<0.0001
Anthropometric and blood pressure measurement			
BMI (kg/m^2^)	28.34 ± 4.4	28.82 ± 4.2	0.427
Systolic blood pressure (mm Hg)	123 ± 16	126 ± 15	0.208
Diastolic blood pressure /mm Hg)	83 ± 9.0	85 ± 8.2	0.122
Oxidative stress markers			
Lipoperoxides (μmol/L)	0.334 ± 0.05	0.356 ± 0.05	0.005
Superoxide dismutase (U/g Hb)	1.23 ± 0.17	1.18 ± 0.13	0.023
Glutathione peroxidase (U/g Hb)	56.2 ± 16.9	50.3 ± 14.5	0.010
Total antioxidant status (μmol/L)	1020 ± 166	1052 ± 185	0.215
SOD/GPx ratio	0.023 ± 0.007	0.025 ± 0.007	0.061
Antioxidant gap (μmol/L)	292 ± 170	305 ± 191	0.616

Quantitative data show means ± standard deviation. *HDL-c* high density lipoprotein cholesterol, *BMI* body mass index. The *p* value was determined with Student’s t test

### Psychological status in pre- and postmenopausal women

Postmenopausal women have lower self-esteem, 32 (32%, 95% CI: 23-41%) vs. 12 (12%, 95% CI: 6-18%), *p* = 0.001; and lower quality of life: 75 (74%, 95% CI: 66-83%) vs. 59 (60%, 95% CI: 49-68%), *p* < 0.05, compared with premenopausal women. The prevalence of postmenopausal women reporting effects on their physical and social quality of life was 64 (63%, 95%CI: 54-73%) and 67 (66%, 95% CI: 57-76%), respectively, *p* < 0.01 (Table 2).

**Table 2:** Prevalence of women with psychological disturbances and low quality of life, by study group, according the score of tests conducted

Test	Premenopausal women (*n* = 101)	Postmenopausal women (*n* = 101)	*p* value
Coopersmith Self-Esteem Inventory (Low < 50)	12 (12%, 6–18%)	32 (32%, 23–41%)	0.001
Zung Self-Rating Anxiety Scale (Anxiety ≥ 45)	24 (24%, 16–32%)	32 (32%, 23–41%)	0.209
Zung Self-Rating Depression Scale (Depression ≥ 40)	21 (21%, 13–29%)	27 (27%, 18–35%)	0.341
World Health Organization Quality of Life, brief (WHOQoL-brief)			
Total score (Low < 96)	59 (60%, 49–68%)	75 (74%, 66–83%)	0.039
Physical subscale (Low < 26)	41 (41%, 31–50%)	64 (63%, 54–73%)	0.005
Psychological subscale (Low < 23)	56 (56%, 47–66%)	62 (61%, 52–71%)	0.686
Social subscale (Low < 12)	43 (43%, 33–52%)	67 (66%, 57–76%)	0.005
Environmental subscale (Low < 30)	73 (74%, 65–82%)	83 (82%, 75–89%)	0.150

Data show frequency, percentage and 95% confidence interval. The *p* value was determined with chi square test

### Psychological status, quality of life and oxidative stress

**Fig. 1:**
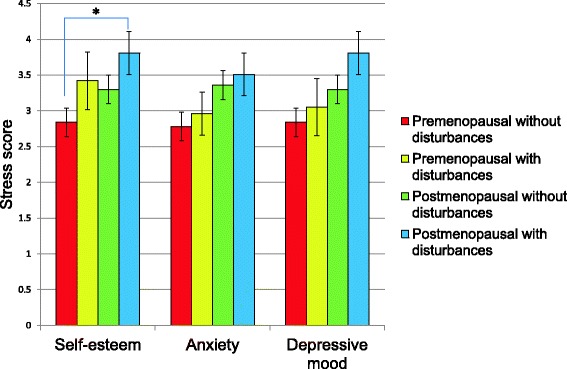
Stress score in the study groups stratified by the different psychological status. The *p* value was determined with ANOVA and Dunnett test as *post hoc*, considering the group of premenopausal women without alterations as control group, *p = 0.01

In the stratified groups, stress scores were higher in postmenopausal women with psychological disturbances in all the tests, compared with premenopausal women without disturbances, but only statistically significant in SEI (Fig. 1).

**Fig. 2:**
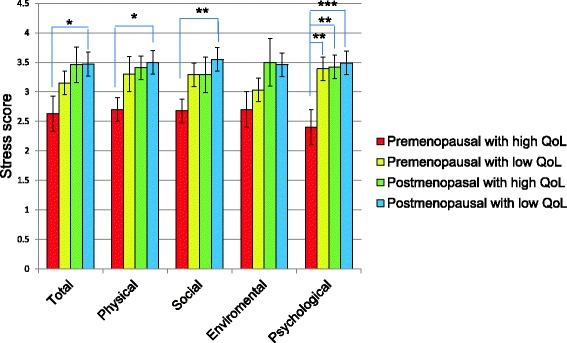
Stress score in study groups, stratified by quality of life and its subscales. The *p* value was determined with ANOVA and Dunnett test as *post hoc*, considering the group of premenopausal women with high quality of life as control group, *p < 0.05, **p < 0.0001, ***p < 0.01. QoL: quality of life

Likewise, we observed that the stress score was higher in postmenopausal women with low quality of life in all WHOQoL-brief subscales, except in environmental subscale, compared with premenopausal women with high quality of life (Fig. 2).

Furthermore, in the univariate analysis, we found a positive correlation between LPO levels and all the tests, except for the self-esteem score, and a negative correlation with the total WHOQoL-brief score in postmenopausal women. In this group, only SEI score and total WHOQoL-brief remained as independent predictors of LPO levels in the multivariate model. Thereby, multiple linear regression analysis revealed that average LPO levels increase by 0.0007 μmol/L for every 1-point increase in the SEI test and by 0.001 μmol/L for every 1-point decrease in the WHOQoL-brief, after to be adjusted for the number of cigarettes consumed, hours of sleep and BMI. Anxiety and dysthymia scores remain positively associated with LPO levels, but not statistically significant; that may be because these are affected by the pro-oxidant factors included in the model; even so, when SAS and SDS increase by 1 point, the LPO levels increase by 0.0004 and 0.0003 μmol/L, respectively (Table 3).

**Table 3:** Univariate and multivariate analysis for the relationship between lipoperoxides levels and the score of the tests conducted in postmenopausal women

	Univariate		Multivariate	
Test	r	*p* value	Unstandardized β (standard error)	*p* value
Coopersmith Self-Esteem Inventory	−0.030	0.385	0.0007 (0.0003)	0.034
Total WHOQoL	−0.298	0.002	−0.001 (0.0005)	0.016
Zung Self-Rating Anxiety	0.256	0.006	0.0004 (0.0005)	0.501
Zung Self-Rating Depression	0.213	0.018	0.0003 (0.0006)	0.593

Data show Pearson correlation coefficient and *p* value, and multiple linear regression parameters, using LPO levels as dependent variable and the scores of tests as independent variables, the model was adjusted by number of cigarettes consumed and hours of sleep by day and BMI as pro-oxidant life-style factors. R = 0.389, R^2^ = 0.151, *p* = 0.036

Similarly, we observed a negative correlation between LPO levels and each of the WHOQoL-brief subscales scores in the same group. In multivariate model, the subscales score remained negatively associated with LPO levels, except psychological score, after adjusting for the same pro-oxidant factors; although only social score was statistically significant. In this model, when the social score decreased by 1 point, average LPO levels increase by 0.010 μmol/L, and when the environmental and physical scores also decrease by 1 point each, LPO levels increase by 0.001 and 0.0003 μmol/L, respectively. (Table 4). There were no correlations between any of the tests and LPO levels in premenopausal women.

**Table 4:** Relationship between lipoperoxides levels and the score of each subscale of the World Health Organization Quality of Life (brief), in postmenopausal women, with univariate and multivariate analysis

	Univariate		Multivariate	
Subscale	r	*p* value	Unstandardized β (standard error)	*p* value
Physical	−0.209	0.020	−0.0003 (0.002)	0.842
Psychological	−0.212	0.019	0.002 (0.002)	0.409
Social	−0.405	<0.0001	−0.010 (0.003)	0.002
Environmental	−0.271	0.004	−0.001 (0.002)	0.409

Data show Pearson correlation coefficient and *p* value, and multiple linear regression parameters using LPO levels as dependent variable and the subscale scores of WHOQoL-brief as independent variables; the model was adjusted by number of cigarettes consumed and hours of sleep by day and BMI as pro-oxidant life-style factors. R = 0.423, R^2^ = 0.179, *p* = 0.012

## Discussion

Menopause occurs earlier in Latin American women compared with women in USA and Europe; therefore, the Latin American women suffer for longer periods [25, 26], affecting their quality of life for longer. The psycho-neuro-endocrine relationship in social adaptation of women in menopause is recognized, since many of the endocrine changes affect self-esteem and mood states during the menopausal transition [27]. In addition, it has been described that the climacteric symptoms are directly related to biologic and sociocultural factors that cause low quality of life in postmenopausal women [28–32]. In this study, the proportion of women with low quality of life was different in total WHOQoL and their subscales, except in psychological and environmental scales, between pre- and postmenopausal women, such as self-esteem. These results are not consistent with other publications that found a negative relationship between psychological aspects and postmenopause [33, 34]. In fact, mood changes were equally frequent seen in the study groups using specific tests for anxiety and depression (SAS and SDS), most likely because the participants were community-dwelling, contrary to most studies where the women were recruited from clinical settings. With reference to self-esteem, recent studies found that aging process and menopausal status are not related with self-esteem, although they used different tests [35, 36].

During postmenopausal period, OS is increased, as we previously noted, probably by severe symptoms [10]; thus, it is likely that estradiol exerts its antioxidant action not only through its chemical structure but through its influence on natural cellular antioxidant enzyme activity, via intracellular signaling cascades and by up-regulation of the expression of antioxidant genes [37, 38], therefore the postmenopausal women possess reduced antioxidant capacity that induces high lipoperoxide levels. In this study, we invited other women and increased the sample size, and confirmed our previous findings. Moreover, as we noted above, depressive mood and anxiety are considered pro-oxidant factors, and cause an increment of OS [12, 13]; therefore, the aim of this study was to establish the relationship between the OS with psychological disturbances and quality of life in the postmenopausal period. In this regard, we sought the relationship between each subscale of WHOQoL and other tests that evaluated mood changes and self-esteem to establish whether the intensity of postmenopausal discomfort increased OS.

Among the various functions of estrogens, one of the most important is their role in the functional integrity of the central nervous system [9, 39]; hence, when gonadal function (estrogen production) begins to decline in women, there is an increase in alterations that cause OS, such as depressive mood, anxiety and insomnia. When we stratified by psychological disturbances and menopausal status, we observed that postmenopausal women with anxiety or depressive mood had higher stress score than premenopausal women without mood changes. Corroborating the evidence that those psychological disturbances produce OS, we showed a positive relationship between the scores of the tests used in this study and lipoperoxide levels in a multivariate model. In relation to anxiety, Bouayed et al. (2009) [40] pointed out a link between OS and high-anxiety-related behavior, confirming our findings; however, the analyzed studies in the literature review did not explain the underlying mechanisms. Some studies have indicated an association between OS and psychological depressive symptoms in females, and shown a potential link between depression and cancer due to oxidative DNA damage via neutrophil activation [12, 41–43].

By associating the changes in quality of life and self-esteem with OS, we found that the postmenopausal women with low quality of life or self-esteem have high OS, measurement by stress score, and when we used lipoperoxide levels as OS marker, remains only the association with quality of life during postmenopause, probably because the change in the levels of this marker is higher than the other markers after menopause. This relationship is reflected in the physical, social and environmental subscales of the WHOQoL. In this sense, this test establishes the improvement of the quality of life [24]; that is why negative correlations were observed in the multivariate model. Additionally, psychological alterations in the WHOQoL questionnaire were associated with OS in univariate analysis, corroborating the findings with tests that determine anxiety and dysthymia; although in multivariate analysis this relationship is lost.

With regard to the social and environmental dimensions of the WHOQoL, we observed a negative correlation between LPO levels and subscale scores in the postmenopausal women. We were not able to find reports in the literature that describe associations between dimensions social and environmental and OS; but some authors suggest that some alterations related to menopause, such as vasomotor symptoms, causes quality of life changes [27] and the psychological symptoms are related to the life events. Life events, family dysfunction and poor social support are important modulators of menopausal symptoms, and the severity of postmenopausal symptoms has been correlated to how problems are handled, suggesting that vulnerability to stress contributes to worsening of menopausal symptoms [44, 45]. Then, the findings of our study suggest that postmenopausal psychological changes alter their social and environmental aspects of quality of life producing an increase of OS.

Also, our results show that the postmenopausal women with low self-esteem had high stress score compared with premenopausal women with high self-esteem.

As we noted, during menopause many women experience negative symptoms that affect their quality of life and, therefore, their feelings as well as their self-esteem. Indeed, health status after the menopause is associated with feelings of body shame and lower psychological well-being compared with health status during the reproductive stage [46], these feeling of shame and lower psychological well-being causes an increase in OS.

Finally, the potential impact of the perception of quality of life and self-esteem on OS has not previously investigated; therefore, the importance of this work is that it shows that psychological changes, low quality of life and low self-esteem cause an increase in OS. It is important to consider that it is a cross-sectional study and therefore the sample may not be representative; then, it is necessary to carry out prospective studies with representative samples to confirm our findings.

## Conclusion

Our findings suggest that oxidative stress is increased in postmenopausal women with dysthymia, anxiety and low quality of life, may be associated to estrogens depletion; hence it is likely that if women with psychological alterations are put under hormone therapy with estrogen or another relief treatment, their quality of life and self-esteem will improve and, therefore, decrease their OS.

## Abbreviations

95% CI: 95% confidence interval
BHT: Butylated hydroxytoluene
BMI: Body mass index
FSH: Follicle stimulant hormone
GAP: Antioxidant gap
GPx: Glutathione peroxidase
HDL-c: High density lipoprotein cholesterol
LPO: Lipoperoxide levels
OS: Oxidative stress
QoL: Quality of life
ROS: Reactive oxygen species
SAS: Zung self-rating anxiety scale
SDS: Zung self-rating depression scale
SEI: Coopersmith self-esteem inventory
SOD: Superoxide dismutase
TAS: Total antioxidant status
TBARS: Thiobarbituric acid reacting substances
WHOQoL-brief: World Health Organization Quality of Life, brief
